# Gallstone ileus: a rare type of intestinal obstruction in Ghana

**DOI:** 10.4314/gmj.v55i1.13

**Published:** 2021-03

**Authors:** Offei K Asare, Henry E Obaka, Nelson K Affram

**Affiliations:** 1 Department of Surgery, Korle-Bu Teaching Hospital, Accra, Ghana; 2 Department of Surgery, Volta Regional Hospital, Ho, Ghana

**Keywords:** Gallstone, Ileus, Intestinal Obstruction, Cholelithiasis, Gall bladder

## Abstract

Gallstone ileus is an uncommon cause of intestinal obstruction in Ghana. A patient with gallstone ileus that was diagnosed intra-operatively after being treated conservatively as adhesive small bowel obstruction for ten days is reported to highlight the importance of the clinical information in this condition. A 60-year-old Chinese male with recurrent intestinal obstruction who was initially being managed at a primary healthcare facility was subsequently referred to a tertiary hospital on account of computer tomography diagnosis of an intraluminal object obstructing the bowel. He had hypokalaemia that was corrected before surgery. Intra-operatively, a 4.5cm in diameter gallstone was removed from the terminal ileum. Gallstone ileus is rare in Ghana. A high index of suspicion is required to avoid a delay in diagnosis due to the low incidence of cholelithiasis in Ghana.

**Funding:**

None declared

## Introduction

Gallstone ileus refers to mechanical obstruction of the bowel by a gallstone. It is estimated to contribute about 1–4% of mechanical intestinal obstruction world-wide; affecting females more than males.^1^–^3^ Gallstone ileus occurs in individuals with previous history of gallstones. It is associated with increased morbidity and mortality due to its high frequency in the elderly, who frequently have co-morbidities. Gallstone ileus is exceedingly rare in Ghana with an unknown incidence.

Pressure necrosis and erosion by the gallstone causes a biliary-enteric fistula commonly between the gall bladder and duodenum through which the stone migrates into the small bowel. The behavior and direction of the stone give rise to different clinical presentations of the disease. Smaller stones are passed in the stools but impaction occurs at the narrowest portion of the terminal ileum, when the stone is large. A proximal migration of the stone into the first part of the duodenum to obstruct it results in gastric outlet obstruction; the Bouveret syndrome.^4^,^5^,^6^ Erosion of the stone into the colon, usually the transverse colon and less frequently the sigmoid, to obstruct it (gallstone coleus) has also been reported.^7^,^8^

The diagnosis of gallstone ileus can be suspected in patients with previous history of gallstones who present with intestinal obstruction. A computerized tomography scan of the abdomen is the best confirmatory diagnostic tool.^9^ Following adequate resuscitation, a laparotomy with enterotomy and stone retrieval is therapeutic. Laparoscopic enterolithotomy is safe and effective in the treatment of Bouveret syndrome.^10^ Cholecystectomy and repair of fistula is done as a two-stage procedure in order to avoid mortality and reduce morbidity in the elderly with significant co-morbidities who are mostly affected.^11^

We report a case of gallstone ileus that was diagnosed during surgery and highlight all relevant clinical information that were present in this case to draw the attention of clinicians to this uncommon condition that could appear in our practice.

## Case Report

A 60-year-old Chinese male was referred to a tertiary hospital on account of small bowel obstruction suspected to be due to post-operative adhesions from previous appendectomy. He presented to a primary healthcare facility eight days earlier with recurrent colicky abdominal pains, absolute constipation, copious bilious vomiting and abdominal distension.

The patient was admitted and managed conservatively for eight days with intravenous fluids and broad-spectrum intravenous antibiotics.

**Figure 1:**
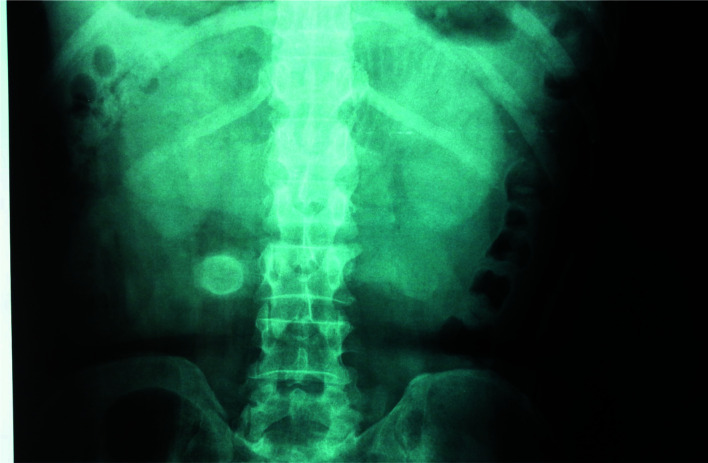
Plain x-ray showing radio-opaque object in the right lower quadrant of the abdomen.

He experienced brief periods of remissions of symptoms characterized by passage of flatus, resolution of abdominal distention, abolition of pain and absence of nasogastric drainage usually over 24 hours. Plain abdominal X-rays showed dilated loops of jejunum and a well-defined spherical opaque object in the right iliac fossa (Figure 1).

This heightened our suspicion of a foreign body in the lumen of the bowel and also the possibility of calcification around a retained gauze after a previous appendectomy. The object had a radio-opaque outer shell which contrasted clearly with the inner core which was radiolucent. There was no obvious pneumobilia.

**Figure 2:**
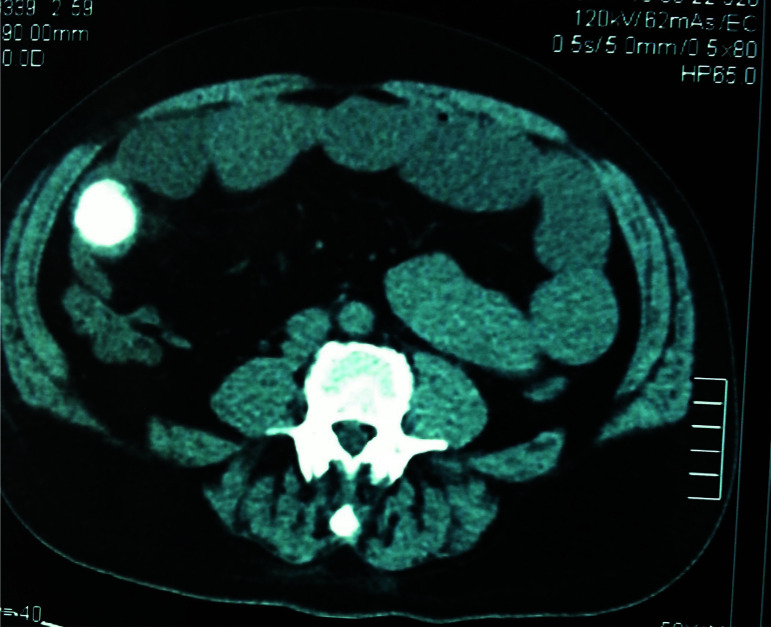
CT scan showing gallstone in the right lower quadrant of the abdomen.

His symptoms had resolved on arrival at the tertiary hospital but recurred and progressively worsened over a 24-hour period. Additional history obtained was that he had cholelithiasis with previous episodes of gallstone colics. An abdominal CT-scan done at the primary hospital showed dilated loops of small bowel and a well-defined radio-opaque spherical object in the right lower quadrant in the lumen of a bowel (Figure 2).

Gallstones were not identified in the gallbladder. Emergency laparotomy was indicated on account of acute intestinal obstruction, with a working diagnosis of an impacted gallstone or a migrated gossypiboma into the bowel. An exploratory laparotomy was scheduled.

A nasogastric tube was passed to decompress the abdomen. He was adequately rehydrated with crystalloids while the urine output was monitored with an indwelling urethral catheter. His registration serum electrolytes revealed hypokalaemia of 2.8mmol/L which was corrected with IV potassium chloride to 3.7mmol/L over a 24-hour period. His hemoglobin concentration was 14.6g/dl. Ceftriaxone and metronidazole infusions were started and he underwent surgery with ASA score of IIIE.

**Figure 3:**
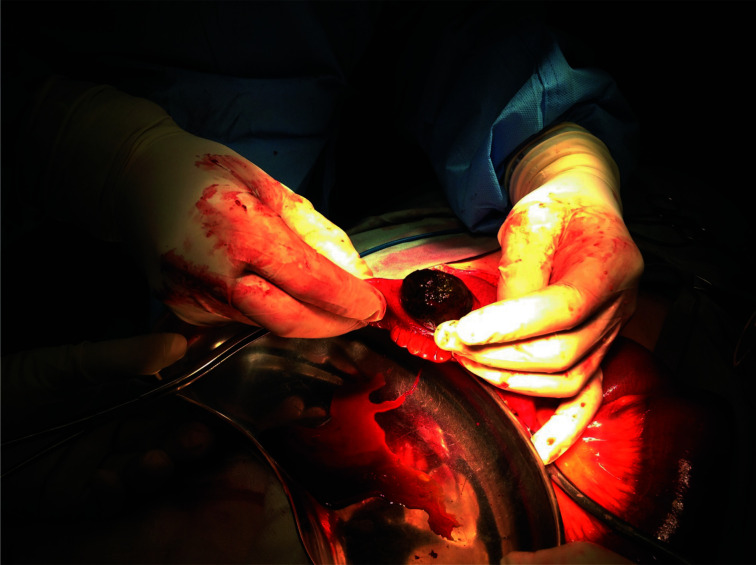
Enterotomy and retrieval of gallstone from the terminal ileum.

Findings at surgery were a 4.5cm in diameter gallstone (Fig. 3) impacted in the ileum about 15cm from the ileocaecal junction with collapsed distal ileum and colon, and distended proximal small bowel.

The stone was milked back and enterotomy and retrieval of stone with primary closure of small bowel was done. He was discharged on the 8^th^ post-operative day following uneventful recovery. Cholecystectomy was not done for this patient on account of dense adhesions on the undersurface of the liver involving the biliary tract area. He was scheduled for review in six weeks but was lost to follow up.

## Discussion

Gallstone ileus is an uncommon cause of intestinal obstruction making it the least suspected differential diagnosis when a patient presents with mechanical bowel obstruction in Ghana. As a result, the diagnosis is usually delayed and made at surgery. During the 8 days that this patient was in a primary health facility he experienced intermittent episodes of small bowel obstruction, the result of the stone being dislodged occasionally along its path. These episodes were interpreted as intermittent resolution of small bowel obstructions from adhesions until a plain abdominal X-ray was taken. The stone was evident on the plain radiograph but with inadequate clinical information, the diagnosis of gallstone was not considered; gossypiboma and calcified bezoars which are not uncommonly encountered surgical pathologies were what could be imagined due to the presence of the calcified foreign body on the plain X-ray.^12^

In a retrospective study over a 21-year period in Spain, only 30% of cases managed were diagnosed preoperatively.^13^ This is consistent with several other earlier studies which estimated the preoperative diagnosis to be 10–44% hence underscoring the need for a high index of suspicion in the diagnosis of gallstone ileus.^14^

The plain X-ray of this patient showed a radio-opaque spherical object close to the right iliac crest. However, pneumobilia was not obvious on the film (Fig 1). It was obvious on the CT scan that this object was in the lumen of the small bowel (Fig 2). It was certain that he had a complete obstruction from a foreign body and needed urgent surgical intervention. It was not until retrieval of a gallstone at surgery that a firm diagnosis was made (Fig 3). It should be emphasized that, in a clinical setting without the availability of a CT scan, the decision to intervene surgically must be based on a good history and physical examination that point to the need for urgent surgery, since plain x-rays have significant limitations in the diagnosis of gallstone ileus. Awareness of this uncommon condition must be created among clinicians in Ghana and the sub-region since it is one of the uncommon pathologies encountered in surgery. However, the patient being a Chinese, where cases of gallstone ileus have been reported should aid early diagnosis.^15^,^16^ The absence of gallstone in the gall bladder on imaging signifies a patent biliary enteric fistula which allowed stones to pass into the small bowel and one should look for pneumobilia to confirm the diagnosis. If the fistula does not close, there remains about 22% risk of developing recurrent gallstone ileus in the future.^11^,^17^

CT scan is superior to plain X-rays in the diagnosis of gallstone ileus.^2^ Contrast enhanced CT scan increases the sensitivity and specificity of diagnosis. This is because only about 10% of gallstones are calcified enough to be visualized by plain X-rays.^2^ The pattern of calcification noted in the stone on the X-ray could be the result of deposition of calcium substances from the bowel luminal content onto the stone as it rolled slowly along the length of the small bowel; snow balling effect.

This could have resulted in the stone gaining enough size (4.5cm) to obstruct the bowel at its narrowest part with a diameter of 2cm.^14^ Otherwise the stone, like other solitary stones in the gallbladder with diameter less than two centimeters would have passed through the bowel and be expelled without consequence.^18^,^19^

The stone was retrieved through an enterotomy which is the recommended and safest procedure for many of these patients due to their age and the associated comorbidities. 1,2,4,8 Under patient optimal conditions, a one-stage procedure which involves enterotomy and retrieval of stone, repair of fistula and cholecystectomy is performed. The intra-operative anatomy of the extra hepatic bile duct area was that of dense adhesions preventing easy access to the gallbladder. A one-stage procedure carried a higher risk of biliary injury and so deferring the second stage procedure of cholecystectomy and repair of the fistula became the most appropriate option.

In the background of lack of consensus on the best time to treat the fistula, with authors divided between one and two-stage approaches,^8^,^15^,^20^–^22^ it is however reasonable to attempt treating the fistula at the first surgery. This should be informed by the stability of the patient before and during surgery, absence of co-morbidities as well as intra-operative findings that permit expeditious excision of the gallbladder and closure of the duodenum. Only when this is not possible as noted in this case should a second stage approach be adopted; which if not done leaves the patient with high risk of recurrent cholangitis.

## Conclusion

Gallstone ileus is a rare clinical entity, and a high index of suspicion is required to avoid a delay in diagnosis. A CT scan is the most appropriate tool required for confirming the diagnosis. Enterotomy and retrieval of stone alone is adequate; cholecystectomy can be performed at the same time if patient's condition permits a one-stage procedure.
